# Supplementary material for: Lipoprotein (a) as a Cardiovascular Risk Factor in Controversial Clinical Scenarios: A Narrative Review
Source: Int J Mol Sci. 2024 Oct 14;25(20):11029. doi: 10.3390/ijms252011029 (PMC11507037; doi:10.3390/ijms252011029)
Supplement: Supplementary file 1 [file ijms-25-11029-s001.zip › ijms-3230333-supplementary.pdf]

Article Name	Authors	Year of Publication	Study Aim
		**Atrial Fibrillation**	
Low lipoprotein(a) concentration is associated with atrial fibrillation: a large retrospective cohort study	Tao J, Yang X, Qiu Q, et al.	2022	To elucidate the potential relationship between Lp(a) quantiles and AF.
Lp(a) (Lipoprotein [a]) and Risk for Incident Atrial Fibrillation: Multi-Ethnic Study of Atherosclerosis	Garg PK, Guan W, Karger AB, et al.	2020	To examine the association of circulating Lp(a) levels with incident AF in a multiethnic prospective cohort.
Association of Lipoprotein (a) variants with risk of cardiovascular disease: a Mendelian randomization study	Xia J, Guo C, Liu K, et al.	2021	Estimate the causal association between variants in genes affecting Lp(a) concentrations and CVD in people of Han Chinese ethnicity using Mendelian randomization.
Elevated Lipoprotein(a) and Risk of Atrial Fibrillation: An Observational and Mendelian Randomization Study	Mohammadi-Shemirani P, Chong M, Narula S, et al.	2022	To investigate the role of Lp(a) in AF and whether it is independent of ASCVD.
Association Between Lipoprotein (a) and Risk of Atrial Fibrillation: A Systematic Review and Meta-analysis of Mendelian Randomization Studies	Singh S, Baars DP, Desai R, Singh D, Pinto-Sietsma SJ.	2024	Investigate the association between Lp(a) and AF by conducting a meta-analysis of Mendelian randomization studies.
Causal effects of plasma lipids on the risk of atrial fibrillation: A multivariable mendelian randomization study	Jiang Q, Qin D, Yang L, et al.	2021	To investigate the causal effects of lipid profiles on the risk of AF using Mendelian randomization.
Associations of Lipoprotein(a) Levels With Incident Atrial Fibrillation and	Aronis KN, Zhao D, Hoogeveen RC, et al.	2017	To investigate the association of Lp(a) levels with the incidence of atrial fibrillation and ischemic stroke in the ARIC cohort.

Ischemic Stroke: The ARIC Study			
Paradoxical association of lipoprotein measures with incident atrial fibrillation	Mora S, Akinkuolie AO, Sandhu RK, Conen D, Albert CM., et al.	2014	To provide insight into the paradoxical association of LDL cholesterol with AF by evaluating the relationship of various lipoprotein measures and incident AF.
**In Stent Restenosis**			
Lipoprotein(a) and coronary thrombosis and restenosis after stent placement	Wehinger A, Kastrati A, Elezi S, et al.	1999	To evaluate the association between Lp(a) levels and thrombotic and restenotic events after coronary stent implantation.
Plasma lipoprotein(a) is not a predictor for restenosis after elective high-pressure coronary stenting	Ribichini F, Steffenino G, Dellavalle A, et al.	1998	To assess the role of plasma lipoprotein(a) as a predictor for restenosis after elective coronary stenting.
Does lipoprotein (a) level have a predictive value in restenosis after coronary stenting?	Khosravi A, Pourmoghaddas M, Ziaie F, et al.	2011	To determine the predictive value of Lp(a) levels for restenosis after coronary stenting.
Association between baseline lipoprotein (a) levels and restenosis after coronary stenting: meta-analysis of 9 cohort studies	Qin SY, Liu J, Jiang HX, et al.	2013	To assess the relationship between baseline Lp(a) levels and restenosis after coronary stenting.
Impact of high lipoprotein(a) levels on in-stent restenosis and long-term clinical outcomes of angina pectoris patients undergoing PCI with DES in Asian population	Park SH, Rha SW, Choi BG, et al.	2015	To assess the influence of Lp(a) serum levels on the mid-term angiographic and 3-year clinical outcomes following PCI with drug-eluting stents.
Association between plasma lipoprotein(a) concentration and restenosis after stent implantation	Kamitani T, Taniguchi T, Miyai N, et al.	2005	To explore the association between plasma Lp(a) concentration and restenosis after stent implantation.
Lipoprotein (a) is related to In-Stent neoatherosclerosis	Yuan X, Han Y, Hu X, et al.	2023	To analyze the relationship between Lp(a) and in-stent neoatherosclerosis incidence

incidence rate and plaque vulnerability: Optical Coherence Tomography Study			and plaque vulnerability using Optical Coherence Tomography.
Lipoprotein(a) and Long-term In-stent Restenosis after Percutaneous Coronary Intervention	Mahmoud AK, Farina JM, Awad K, et al.	2024	To investigate the association between Lp(a) and ISR.
**Cardiac Allograft Vasculopathy**			
Cardiac allograft vasculopathy: Insights on pathogenesis and therapy	Lee F, Nair V, Chih S.	2020	Review key pathways involved in the pathogenesis and therapeutic approaches for cardiac allograft vasculopathy (CAV).
Lipoprotein(a) and accelerated coronary artery disease in cardiac transplant recipients	Barbir M, Kushwaha S, Hunt B, et al.	1992	To examine the relationship between Lp(a) levels and accelerated coronary artery disease in cardiac transplant recipients.
Lipoprotein(a) levels and heart transplantation atherosclerosis	Chang G, DeNofrio D, Desai S, et al.	1998	To assess the association between Lp(a) levels and accelerated cardiac allograft vasculopathy.
**Bioprosthetic aortic degeneration**			
Role of lipoprotein(a) concentrations in bioprosthetic aortic valve degeneration	Farina JM, Chao CJ, Pereyra M, et al.	2024	To evaluate the association between Lp(a) concentrations and bAV degeneration.
Serum lipoprotein(a) and bioprosthetic aortic valve degeneration	Botezatu SB, Tzolos E, Kaiser Y, et al.	2023	To investigate whether serum Lp(a) concentrations are associated with bioprosthetic aortic valve degeneration.
